# Efficient Hardware Accelerator Design of Non-Linear Optimization Correlative Scan Matching Algorithm in 2D LiDAR SLAM for Mobile Robots

**DOI:** 10.3390/s22228947

**Published:** 2022-11-18

**Authors:** Ao Hu, Guoyi Yu, Qianjin Wang, Dongxiao Han, Shilun Zhao, Bingqiang Liu, Yu Yu, Yuwen Li, Chao Wang, Xuecheng Zou

**Affiliations:** 1 School of Optical and Electronic Information, Huazhong University of Science and Technology, Wuhan 430074, China; 2 Wuhan National Laboratory of Optoelectronics, Huazhong University of Science and Technology, Wuhan 430074, China; 3 School of Mechatronic Engineering and Automation, Shanghai University, Shanghai 200444, China

**Keywords:** 2D LiDAR SLAM, hardware accelerator, Non-linear Optimization CSM

## Abstract

Simultaneous localization and mapping (SLAM) is the major solution for constructing or updating a map of an unknown environment while simultaneously keeping track of a mobile robot’s location. Correlative Scan Matching (CSM) is a scan matching algorithm for obtaining the posterior distribution probability for the robot’s pose in SLAM. This paper combines the non-linear optimization algorithm and CSM algorithm into an NLO-CSM (Non-linear Optimization CSM) algorithm for reducing the computation resources and the amount of computation while ensuring high calculation accuracy, and it presents an efficient hardware accelerator design of the NLO-CSM algorithm for the scan matching in 2D LiDAR SLAM. The proposed NLO-CSM hardware accelerator utilizes pipeline processing and module reusing techniques to achieve low hardware overhead, fast matching, and high energy efficiency. FPGA implementation results show that, at 100 MHz clock, the power consumption of the proposed hardware accelerator is as low as 0.79 W, while it performs a scan match at 8.98 ms and 7.15 mJ per frame. The proposed design outperforms the ARM-A9 dual-core CPU implementation with a 92.74% increase and 90.71% saving in computing speed and energy consumption, respectively. It has also achieved 80.3% LUTs, 84.13% FFs, and 20.83% DSPs saving, as well as an 8.17× increase in frame rate and 96.22% improvement in energy efficiency over a state-of-the-art hardware accelerator design in the literature. ASIC implementation in 65 nm can further reduce the computing time and energy consumption per scan to 5.94 ms and 0.06 mJ, respectively, which shows that the proposed NLO-CSM hardware accelerator design is suitable for resource-limited and energy-constrained mobile and micro robot applications.

## 1. Introduction

SLAM (Simultaneous Localization and Mapping) has been widely used in robots to solve the problem of localization and navigation in unknown environments [1,2,3]. According to the type of sensor, SLAM is classified into visual SLAM and LiDAR SLAM, while according to the dimension of exploration space, SLAM is divided into 2D SLAM and 3D SLAM. Compared with visual SLAM, LiDAR SLAM has higher precision and reliability. The 2D LiDAR SLAM has the advantages of low cost and simple system structure compared with 3D LiDAR SLAM, and it is sufficient for the localization and mapping of robots in indoor and a small range of outdoor scenarios. As for complex environments, geometric information, such as conic features from the 2D LiDAR information, can be extracted and used to build the map more accurately [4], and sensors such as odometer and IMU (Inertial Measurement Unit), in robots, can be added to improve the accuracy of localization and mapping [5]. Therefore, 2D LiDAR SLAM is suitable for energy-constrained and size-limited intelligent mobile robot autonomous navigation [4,5,6].

**Figure 1:**
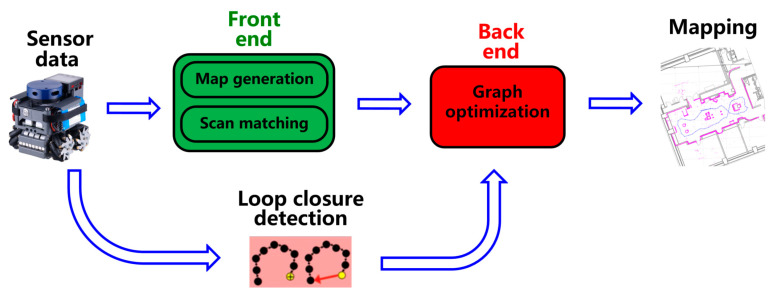
Framework of classic LiDAR SLAM system.

Figure 1 presents a classic LiDAR SLAM system framework, which consists of four major parts: (i) the frontend part locates the robot according to the environmental information collected by the sensor; (ii) the backend part mainly performs map and pose optimization; (iii) the loop closure detection part refers to detecting whether the map information can be closed to reduce the drift of the map and ensure the global consistency of the map; (iv) the final mapping part is the generation and maintenance of map information. In a LiDAR SLAM system, the real-time localization of the intelligent robot itself is the premise of mapping. As one of the key tasks in the localization, scan matching obtains the current pose of the mobile robot by matching the current LiDAR frame with the past multiple frames or maps, which is computationally intensive, time-consuming, and power-hungry.

There are three major scan matching algorithms of 2D LiDAR SLAM: filter algorithm, Non-linear Optimization (NLO) algorithm, and Correlation Scan Matching (CSM) algorithm. The filter algorithm includes the classical filter algorithm and particle filter algorithm. The computational complexity of the classical filter algorithm increases quadratically with the increase in the environment mapping scale. In addition, due to the difficulty of feature extraction and data association, it is easy to cause oscillation and divergence in the iterative process of the filter algorithm, which is a drawback to be dealt with [7,8]. Similarly, the particle filter algorithm also has the same shortcoming, i.e., when the environmental map gets larger, the number of particles increases to meet the positioning and mapping on the larger map; therefore, the computational complexity and resource consumption also increase significantly [9,10,11]. The NLO algorithm transforms the matching problem into a least square problem, which is solved by the Gauss–Newton Method, but it is also easy to fall into divergence and requires a more accurate initial LiDAR pose. In contrast, the CSM algorithm uses the current LiDAR frame and a few historical multi-frames to match within a range where the best pose may exist, and it evaluates the scores of different poses to solve the global optimal solution, which not only avoids the situation of oscillation but also reduces the computation over time. Therefore, the CSM algorithm has been widely used in 2D LiDAR SLAM for various scenarios [12]. However, the way that the CSM algorithm evaluates all poses within a space range still involves a huge amount of computation, imposing a stringent requirement for high computing power for performing real-time SLAM in mobile robot applications.

In the literature of CSM algorithms, the Karto SLAM [13] uses brute force search for scan matching, but it needs high computing-power hardware that results in high hardware overhead and significant energy consumption. In [14], S. Kohlbrecher et al. use an NLO algorithm to improve the scan matching accuracy, but this approach makes it easy to fall into the local optimal solution, and it also requires high frame-rate LiDAR. W. Hess et al. combine the depth-first search, an improved CSM, and an NLO algorithm to get a higher matching accuracy, but it still requires a high-performance computing platform to achieve real-time [15]. In summary, most existing scan matching algorithms have achieved high-performance scan results at the cost of high computational complexity that results in high-computation hardware resources and significant energy consumption.

To solve the issues of high computing power and energy consumption, researchers have started to develop hardware accelerator designs for the existing CSM algorithms recently. K. Sugiura et al. present an FPGA (Field Programmable Gate Array)-based hardware accelerator design that has implemented the conventional CSM algorithm on the programmable logic part by conducting several architectural and algorithmic optimizations to fully exploit the inherent parallelism, which shows real-time performance and high accuracy in typical indoor scenarios [16]. However, the hardware overhead of the CSM core is quite large, and the system power consumption is as high as 2.3 W. M. Bao et al. present a heterogeneous multi-core SoC (System on Chip) implementation of a Real-time Impact-Aware CSM (RIA-CSM) [17]. The most time-consuming part of the CSM algorithm is mapped to an FPGA-based CSM accelerator, while the rest are realized by the quad-core processor so that both the real-time performance and robust localization have been improved significantly. However, similar to the hardware accelerator design in [16], both the hardware overhead and system power consumption of 2.13 W are high. This is because both the CSM hardware accelerator designs suffer high complexity of the CSM algorithms and lack efficient hardware architecture design methods or techniques. In summary, the existing scan matching hardware accelerator designs have not effectively addressed the design challenges of limited hardware resources and constrained power budgets in mobile and micro robot applications.

In this paper, a combination of the NLO algorithm and CSM algorithm, i.e., the NLO-CSM algorithm, is adopted to perform a two-step scan match to address the high computation and easy to fall into divergence issue while ensuring a high scan match accuracy. The conventional CSM algorithm is used as a coarse match to acquire a rational initial pose. The NLO algorithm is then used as a fine match to perform Gaussian–Newton iteration for obtaining an optimal pose. In addition, an efficient hardware accelerator design of the NLO-CSM algorithm is proposed to solve the aforementioned issues of high hardware overhead and energy consumption in the existing works. The major contributions of this paper are as follows:

(1) For the issue of the high computational cost and resource cost of the conventional CSM algorithm, a two-step NLO-CSM algorithm is adopted in this paper. The CSM algorithm performs scanning and matching on a down-sampling low-resolution map to reduce computation. Based on a good initial pose found by the first-step CSM algorithm, the second-step NLO algorithm performs iterative operations to obtain the optimal pose. The optimized NLO-CSM algorithm not only avoids the high computational complexity of brute force searching on the grid map in the conventional CSM algorithm but also reduces computation time and energy consumption of computing hardware, as required. The optimized NLO-CSM algorithm can achieve good scan match performance by avoiding the divergence caused by poor initial pose while performing the NLO algorithm only.
(2) This paper also presents a comprehensive algorithmic analysis of the adopted NLO-CSM algorithm. A corresponding efficient hardware accelerator design is proposed, based on the analysis, to accelerate the major computation-intensive tasks in the NLO-CSM algorithm. By exploiting the algorithm similarity and operator sharing between the two-step algorithm computations, module reusing technique is adopted to further reduce the hardware overhead, as required by the computation of the two-step NLO-CSM algorithm. In addition, pipeline processing strategy is adopted to realize fast computing, therefore achieving high energy efficiency. The algorithmic analysis and corresponding hardware design provide a practical reference for efficient hardware design of scan matching algorithms.
(3) Systematic hardware evaluation, based on both FPGA and ASIC (Application Specific Integrated Circuit) implementations of the proposed NLO-CSM hardware accelerator, has been done. Comparisons among the CPU software solution, FPGA-based hardware accelerator, and ASIC-based hardware accelerator have been carried out to prove the effectiveness of the proposed work, in terms of computing speed and energy efficiency improvements, against existing state-of-the-arts.

The remainder of this paper is organized as follows: Section 2 provides an analysis of the conventional CSM algorithm and NLO-CSM algorithm. Section 3 describes the architecture of the proposed NLO-CSM hardware accelerator. Section 4 presents the implementation results. Section 5 concludes this paper.

## 2. Algorithm Analysis of NLO-CSM

### 2.1. Conventional CSM Algorithm

**Figure 2:**
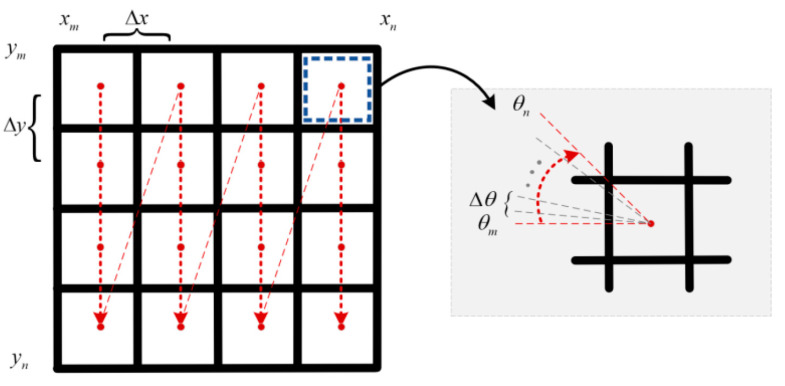
Search space for scan matching in CSM algorithm.

CSM algorithm is a scan matching algorithm, based on occupancy grid maps, which is robust in feature-rich environments. In the conventional CSM algorithm, the system would use brute force search in the space range to get the optimal pose of the LiDAR sensor (i.e., a global optimal solution of the computing observation model). It establishes occupancy grid maps, and each grid represents one pose of LiDAR sensors. In the space range that may have the best matching pose, the CSM algorithm maps the currently scanned frame to the occupancy grid map under different poses and estimates the score according to the fit between the scanned frame and the grid map. The range of search space for the best matching pose estimation can be provided by sensors such as odometers, as shown in Figure 2.

**Figure 3:**
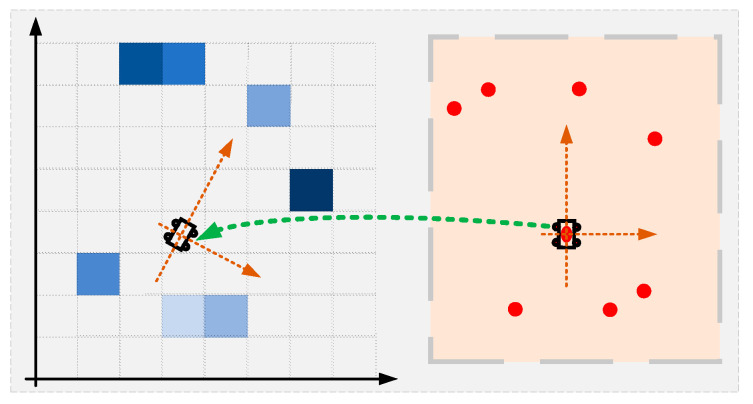
Mapping between scan points and grid map under a LiDAR pose.

Figure 3 shows the mapping between scan points and grid map under a LiDAR pose, illustrating how the scan points are mapped to the occupancy grid map that contains values of estimating probability occupancy by grids. As depicted in Equation (1), the pose of the LiDAR sensor in the world coordinate system is represented by *L* = (*l_x_, l_y_, l_θ_*), and *S_i_*(*L*) is the coordinate *S_i_* of the *i_th_* LiDAR point in the world coordinate system under the pose *L*. The sum of these grids’ probability (i.e., *M_i_*[*S_i_*(*L*)]) is considered as the score under the current sensor pose, as depicted in Equation (2). By this means, the whole local space can be searched to get the optimal pose with the maximal score [13].

(1) $$S_{i}\left(L\right)=\left(\begin{array}{cc} \cos l_{\theta } & -\sin l_{\theta }\\ \sin l_{\theta } & \cos l_{\theta } \end{array}\right)\left(\begin{array}{c} s_{i,x}\\ s_{i,y} \end{array}\right)+\left(\begin{array}{c} l_{x}\\ l_{y} \end{array}\right),L={\left(l_{x},l_{y},l_{\theta }\right)}^{T}$$

(2) $$score=\sum_{i=1}^{n}M_{i}\left[S_{i}\left(L\right)\right]$$

The pseudocode of the conventional CSM algorithm is shown in Algorithm 1. The function ***get_score*** finds the sum of the grids’ probability as the score under the current sensor pose, and the function ***CSM*** corresponds to the aforementioned space-searching process. It is worth mentioning that the best matching pose calculated by the CSM algorithm can only be expressed by a grid, so the center of the grid is generally used as the position information in the pose. The angles are generated in increments of the step size, so the angle accuracy in the pose is limited by the angle step size. When a higher-precision pose is required, it is necessary to improve the precision of the grid map and reduce the step size of the angle. In general, the required data amount increases quadratically with the precision of the grid map, and the number of poses for the calculated scores also increases, which will greatly increase the map’s memory overhead. Meanwhile, the brute force search requires high-performance general computing platforms, such as multi-core CPU or GPU, which are very power-hungry and not suitable for mobile and micro robots with the requirement of limited resources and constrained power budget.
**Algorithm 1: Conventional CSM Algorithm**1:**Function*****CSM*** (*Map, x_m_, x_n,_ y_m_, y_n,_ θ_m_, θ_n_*)2: *max*_*score* = 0, *best*_*pose* = (*x_m_, y_m_, θ_m_*),3: **for**
*x_i_ in* [*x_m_, x_n_*]4:  **for**
*y_i_ in* [*y_m_, y_n_*]5:   **for**
*θ_i_ in* [*θ_m_, θ_n_*]6:    *score* = ***get_score*** (*Map*, *L_i_*)*, L_i_ =* (*x_i_, y_i_, θ_i_*)7:    **if**
*score* > *best*_*score*
**then**
*best*_*pose* = *L*, *max*_*score* = *score*8:   **end for**9:  **end for**10:**end for**11:**return***best*_*pose*12:**Function*get_score***(*Map*, *L_i_*)*, L* = (*x_i_, y_i_, θ_i_*)13: *θ_i_* → sin *θ_i_*, cos *θ_i_*, *Scan*_*points =* [*s_0_*, *…s_i_*, *…s_k_*]*, s_i_ =* (*s_i,x_*, *s_i,y_*), *score* = *0*14: **for**
*s_i_*
**in** [*s_0_, …s_i_, …s_k_*]15: 
$S_{i}(L)=\left(\begin{array}{cc} \cos L_{\theta } & -\sin L_{\theta }\\ \sin L_{\theta } & \cos L_{\theta } \end{array}\right)\left(\begin{array}{c} s_{i,x}\\ s_{i,y} \end{array}\right)+\left(\begin{array}{c} L_{x}\\ L_{y} \end{array}\right),L={\left(L_{x},L_{y},L_{\theta }\right)}^{T}$
16: *score* = *score* + *Map*(*S_i,x_, S_i,y_*)17:**end for**18:**return***score*

### 2.2. NLO-CSM Algorithm

In this paper, an NLO-CSM algorithm is adopted for low power consumption, fast computing, and high area efficiency. Algorithm 2 shows the pseudocode of the NLO-CSM algorithm. The algorithm consists of two parts: coarse match and fine match. The coarse match process is the same as the aforementioned conventional CSM algorithm, which is used to determine the local range of the best matching pose on a low-resolution occupancy grid map. Note that the low-resolution occupancy grid map is obtained by a down-sampling of the original grid map, with the max pooling method, by a factor of *SR*. The NLO algorithm, as the fine match process of NLO-CSM, transforms the scan matching problem into a least squares problem, which is used to determine the specific position of the best matching pose on the original grid map. The fine match process is the solution of a least-square formula solved by the Gauss–Newton method. By this means, the NLO algorithm gets the step of the pose Δ*L* for each iteration and updates the pose for the next iteration, and the iteration stops when the matching accuracy is reached, as shown in the function ***Fine_match*** in Algorithm 2. It should be noted that the core of the pose update process of the fine match is the calculation of the Hessian matrix H and the residual matrix K implemented by the function ***get_HessianDerivs*** in Algorithm 2.

**Figure 4:**
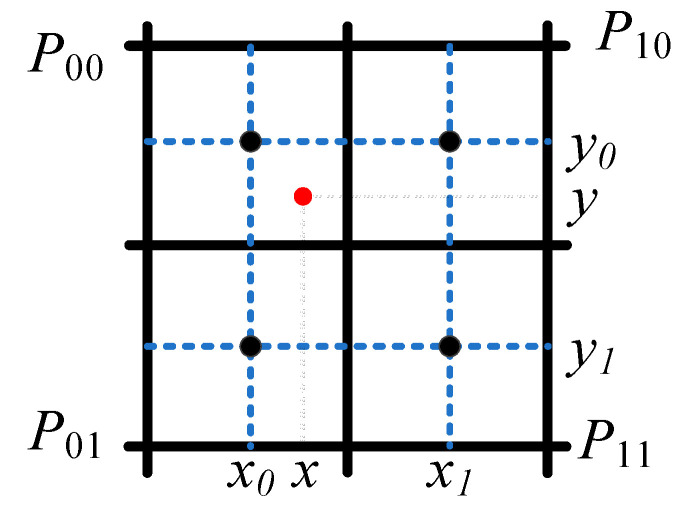
Schematic diagram of the bilinear interpolation.

The coarse match process on the low-resolution grid map provides a rational initial pose for the non-linear optimization algorithm in the fine match process with a lower calculation amount. Thus, in order to improve the overall matching accuracy of the algorithm, the pose provided by the coarse match process can avoid the local optimum and the non-convergence caused by a poor initial pose. The fine match process utilizes this initial pose to perform bilinear interpolation on the original grid map for better fitting and to iteratively solve for the optimal pose. Figure 4 shows the schematic diagram of the bilinear interpolation, while Equations (3) and (4) depict the interpolation method. *Pm* corresponds to the map interpolation of *Map* and *Si*.

(3) $$M\left(P_{m}\right)\approx \frac{y-y_{0}}{y_{1}-y_{0}}\left(\frac{x-x_{0}}{x_{1}-x_{0}}M\left(P_{11}\right)+\frac{x_{1}-x}{x_{1}-x_{0}}M\left(P_{01}\right)\right)+\frac{y_{1}-y}{y_{1}-y_{0}}\left(\frac{x-x_{0}}{x_{1}-x_{0}}M\left(P_{10}\right)+\frac{x_{1}-x}{x_{1}-x_{0}}M\left(P_{00}\right)\right)$$

(4) $$\begin{array}{c} \frac{\partial M\left(P_{m}\right)}{\partial x}\approx \frac{y-y_{0}}{y_{1}-y_{0}}\left(M\left(P_{11}\right)-M\left(P_{01}\right)\right)+\frac{y_{1}-y}{y_{1}-y_{0}}\left(M\left(P_{10}\right)-M\left(P_{00}\right)\right)\\ \frac{\partial M\left(P_{m}\right)}{\partial y}\approx \frac{x-x_{0}}{x_{1}-x_{0}}\left(M\left(P_{11}\right)\pm M\left(P_{10}\right)\right)+\frac{x_{1}-x}{x_{1}-x_{0}}\left(M\left(P_{01}\right)-M\left(P_{00}\right)\right) \end{array},\nabla M\left(P_{m}\right)=\left(\frac{\partial M\left(P_{m}\right)}{\partial x},\frac{\partial M\left(P_{m}\right)}{\partial y}\right)$$

**Algorithm 2: NLO-CSM Algorithm**
1:*Map* = **matrix**[*p*, *q*], *Scan*_*points =* [*s_0_*, *…s_i_*, *…s_k_*]*, s_i_ =* (*s_i,x_*, *s_i,y_*)//*n* is the number of LiDAR points, *n* = *k*+12: **Function** ***Coarse_match***3: *max*_*score* = 0, *best*_*pose* = (*x_m_, y_m_, θ_m_*)4: **for**
*x_i_ in* [*x_m_, x_n_*]5: **for**
*y_i_ in* [*y_m_, y_n_*]6: **for**
*θ_i_ in* [*θ_m_, θ_n_*]7: *score* = ***get_score***(*L_i_*)*, L_i_ =* (*x_i_, y_i_, θ_i_*)8: **if** *score* > *best*_*score* **then**9: *best*_*pose* = *L*, *max*_*score* = *score*10: **Function *Fine_match*(*L_0_*)**, *L_0_* = (*L*_0,*x*_*, L_0,y_, L_0,θ_*)11: ∆*L* = (∆*L_x_*, ∆*L_y_*, ∆*L_θ_*) = (0, 0, 0), *H* = **0**, *K* = **0**12: **for** *i* **in** [1, *λ*]//*λ* is the number of Gauss-Newton iteration times13: *L_i_* = *L_i_*_-1_+∆*L =* (*L_i_*_-1,*x*_*, L_i_*_-1*,y*_*, L_i_*_-1*,θ*_) + (∆*L_x_*, ∆*L_y_*, ∆*L_θ_*)14: (*H, K*) = ***get_HessianDerivs***(*L_i_*)15: ∆*L* = (∆*L_x_*, ∆*L_y_*, ∆*L_θ_*) = *H*^−1^∙*K*16: **SubFunction *get_score*(*Li*)**, *L_i_* = (*x_i_*, *y_i_*, *θ_i_*)17:*θ_i_* → sin *θ_i_*, cos *θ_i_, score* = *0*18:**for** *s_i_* **in** [*s_0_, …s_i_, …s_k_*]19:

$S_{i}(L)=\left[\begin{array}{cc} \cos\theta_{i} & -\sin\theta_{i}\\ \sin\theta_{i} & \cos\theta_{i} \end{array}\right]\left[\begin{array}{c} s_{i,x}\\ s_{i,y} \end{array}\right]+\left[\begin{array}{c} x_{i}\\ y_{i} \end{array}\right]=\left[\begin{array}{c} S_{i,x}\\ S_{i,y} \end{array}\right]$

20:*score* = *score + Map*(*S_i,x_, S_i,y_*)21:**return** *score*22:**SubFunction *get_HessianDerivs* (*L_i_*)**, *L_i_ =* (*L_i_*_,*x*_*, L_i,y_, L_i,θ_*)23:*θ_i_* → sin *L_i,θ_*, cos *L_i,θ_*24:**for** *i* **in** [1, *k*]25:

$S_{i}(L_{i})=\left[\begin{array}{cc} \cos\theta_{i} & -\sin\theta_{i}\\ \sin\theta_{i} & \cos\theta_{i} \end{array}\right]\left[\begin{array}{c} s_{i,x}\\ s_{i,y} \end{array}\right]+\left[\begin{array}{c} x_{i}\\ y_{i} \end{array}\right]=\left[\begin{array}{c} S_{i,x}\\ S_{i,y} \end{array}\right]$

26:

$\frac{\partial S_{i}(L)}{\partial L}=\frac{\partial S_{i}(L)}{\partial (l_{x},l_{x},l_{\theta })}=\left[\begin{array}{ccc} 1 & 0 & -\sin l_{\theta }\cdot s_{i,x}-\cos l_{\theta }\cdot s_{i,y}\\ 0 & 1 & \cos l_{\theta }\cdot s_{i,x}-\sin l_{\theta }\cdot s_{i,y} \end{array}\right]$

27:

$\nabla M(S_{i}),P_{m}\leftarrow$


$Map\;interpolation(Map,S_{i})$

28:

$J\left[i\right]=\nabla M\left(S_{i}\right)\frac{\partial S_{i}\left(L_{i}\right)}{\partial L_{i}},F\left[i\right]=1-P_{m}$

29: 
**end**
30: $H=J^{T}J$, $K=J^{T}F$

**Figure 5:**
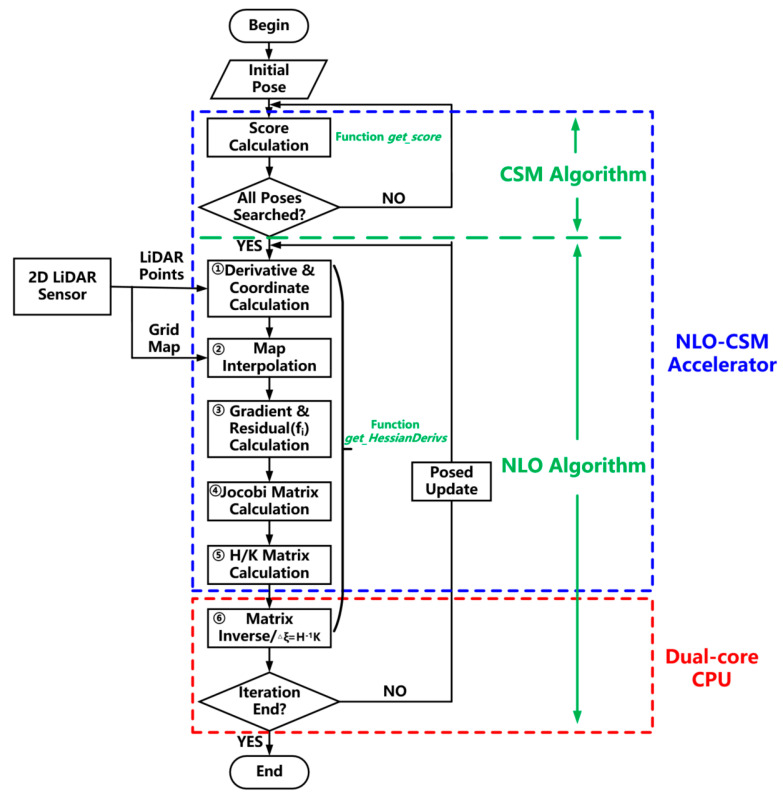
Flow chart of the NLO-CSM algorithm and the corresponding software and hardware design partition.

The corresponding flow chart of the NLO-CSM algorithm and software–hardware design partition is shown in Figure 5. The Score Calculation process corresponds to the CSM-based coarse match process. After all the poses have been retrieved by the coarse match process, the algorithm enters the NLO-based fine match process. The function ***get_HessianDerivs***, calculating the Hessian matrix H and the residual matrix K in Algorithm 2, has been divided into six calculation processes in Figure 5, i.e., from the Derivative and Coordinate Calculation process to the Matrix Inverse process. As shown in Figure 5, the whole coarse match process and H/K matrix calculation process are selected to be implemented in the proposed NLO-CSM hardware accelerator in this study because the computation of two processes is highly repetitive and significantly intensive in the NLO-CSM algorithm, which needs to be executed for each LiDAR point in a LiDAR frame. In contrast, as the processes of matrix inverse and the final pose update are only executed once after the H/K matrix, corresponding to the last LiDAR point of a LiDAR frame, is obtained and, therefore, considering that the hardware utilization of these two processes is not high, they are selected to be implemented by software executed in a dual-core CPU in this study.

**Figure 6:**
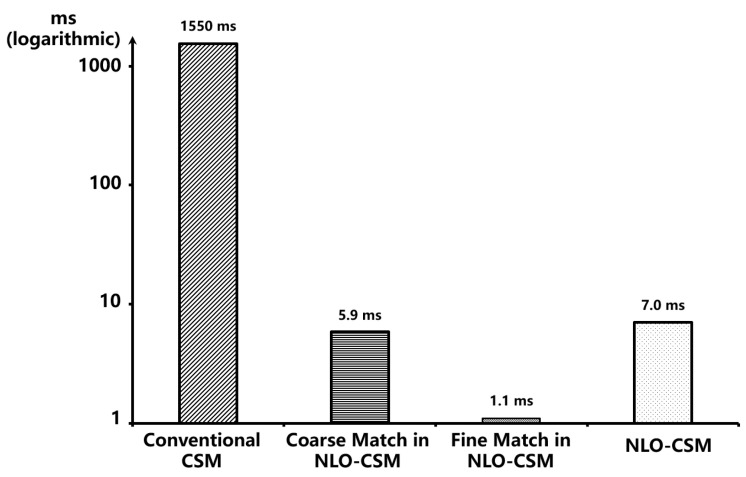
Computation time of the conventional CSM algorithm and the NLO-CSM algorithm based on Intel Core i5-10400 platform.

Figure 6 presents the results of evaluating the amount of computation required by the conventional CSM and NLO-CSM algorithm, on an Intel Core i5-10400 platform, in terms of the computational time as measured. This test is based on an occupancy grid map with a resolution of 5 cm in an 8 m × 15 m indoor scene provided by our collaborator from Shanghai University. The accuracy of used LiDAR is 0.5 mm, the scanning range is 10 m~0.05 m, and the LiDAR frame to be matched contains 90 LiDAR points in this test. The computation time of the conventional CSM algorithm is 1.55 s, as the CSM algorithm needs to match a huge number of poses based on the occupancy grid map. In contrast, the NLO-CSM algorithm only needs about 7.0 ms to complete the overall pose calculation, which is 225 times faster than the CSM algorithm. Therefore, this improvement by the NLO-CSM algorithm is significant, especially for the real-time energy-constrained mobile and micro robot applications.

Table 1 shows the comparison of the localization error between the adopted NLO-CSM and the RIA-CSM published in the latest literature [17]. There are two publicly available datasets that have been used, i.e., the Deutsches Museum dataset and the Revo LDS dataset [15], which have been recorded for testing the Cartographer SLAM system by utilizing the NLO-CSM algorithm for scan matching. The optimal values of the positions and the rotation angles have been verified by the comparison with the results from Cartographer, which incorporates Google’s Ceres, a large-scale nonlinear optimization library, to solve the above nonlinear least square problem [15]. The differences in the position and in the rotation angle, as well as the corresponding standard deviation, are listed in the table for each dataset. It can be seen that the difference in the position by utilizing the adopted NLO-CSM algorithm does not exceed 5 cm, and the difference in the rotation angle does not exceed 0.5 degrees. Even though the difference in the rotation angle of this work, based on the Revo LDS dataset, is a little higher than the RIA-CSM, it should be mentioned that all the localization error types of this work based on the Deutsches Museum dataset are superior to the RIA-CSM, which means a high scan match accuracy has been achieved by the NLO-CSM algorithm.

## 3. NLO-CSM Algorithm Hardware Accelerator Design

### 3.1. Overall System Architecture

**Figure 7:**
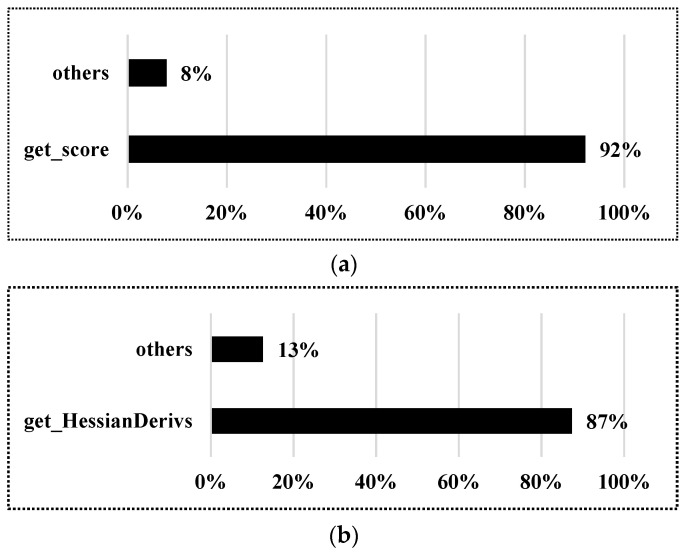
Computational load analysis of major tasks in the NLO-CSM algorithm in: (**a**) coarse match process; (**b**) fine match process.

In order to identify the major computation-intensive tasks in the algorithm for hardware acceleration, a statistical analysis of the pose iteration calculation process in the NLO-CSM algorithm, in Algorithm 2, has been carried out. In this study, considering a typical case of a single-line LiDAR scanning 600 points at a time and 50 times Gauss–Newton iterations on the Intel Core i5-10400 platform, the computation load results of major tasks, by calculating the numbers of addition, shift, and multiplication, are shown in Figure 7. It is found that the main computation-intensive tasks are ***get_score*** and ***get_HessianDerivs***, which take up 92% and 87% of computing resources in the CSM-based coarse match and NLO-based fine match processes, respectively. Notably, according to Algorithm 2, the main calculation operator of ***get_score*** and ***get_HessianDerivs*** is a matrix calculation that is one of the most time-consuming and power-hungry operations. Thus, a hardware accelerator design is proposed to accelerate these two major computation-intensive tasks, which are ***get_score*** and ***get_HessianDerivs*** in the NLO-CSM algorithm, as highlighted in the blue color in Algorithm 2, in this study.

**Figure 8:**
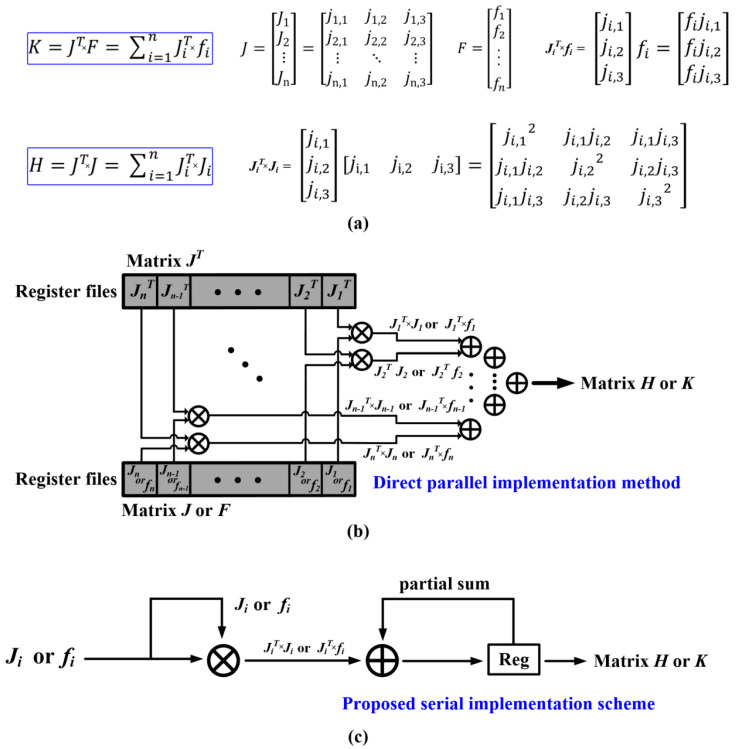
(**a**) Matrix calculation of the H/K matrix; (**b**) direct parallel implementation method; (**c**) proposed serial implementation scheme of the H/K matrix calculation.

Worth noting, the matrix calculation of $H=J^{T}J$ and $K=J^{T}F$ of the ***get_HessianDerivs*** operation in Algorithm 2 of Section 2 could be transformed into $H=\Sigma J_{i}{}^{T}J_{i}$ and $K=\Sigma J_{i}{}^{T}f_{i}$, as shown in Figure 8a. In the direct parallel implementation of the matrix calculation, as shown in Figure 8b, multiple multipliers and a large-scale adder tree are used for calculating the matrix H/K from J and F. When the number of LiDAR points per frame increases, the size of the matrix J and F will become larger, resulting in large memory overhead for storing the matrix J and F. In order to reduce the hardware overhead, the proposed accelerator design uses a serial multiplication scheme to perform multiply-accumulate operations, as described in Figure 8c. In contrast to the parallel method using larger hardware resources of 3*n* multipliers and $3n\left(1-\frac{1}{2^{\left(log_{2}\frac{n}{2}+1\right)}}\right)$ adders (*n* represents the number of LiDAR points), the proposed accelerator design only uses 3 multipliers and 3 adders by performing multiply-accumulate operation for the single *ji* or *fi* once it has been obtained. In terms of the processing speed, the proposed serial implementation scheme uses 6n clock cycles (6 clock cycles for one LiDAR point, which will be introduced later in this section) to complete the matrix multiplication. The direct parallel uses (3+$\frac{15n}{2}$) clock cycles because it takes 6n clock cycles to buffer the input LiDAR frame points (i.e., 90 points in this study) and (3+$\frac{3n}{2}$) clock cycles for the adder tree to perform accumulation.

**Figure 9:**
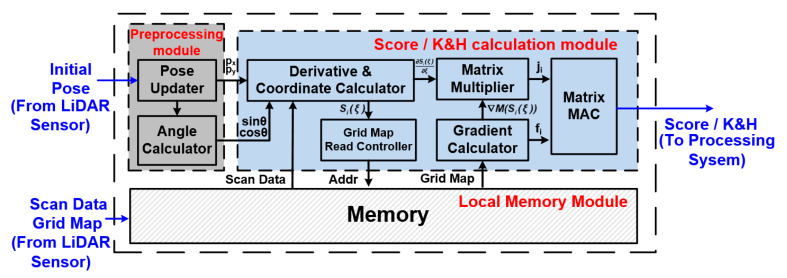
Overall architecture of the proposed NLO-CSM hardware accelerator design.

Figure 9 shows the hardware architecture of the proposed NLO-CSM hardware accelerator design, which consists of three major parts:

(1) The preprocessing module performs the storage update of the pose, as well as the input-data processing of the pose angles, i.e., the sinθ and cosθ calculation, in the ***get_score*** and ***get_HessianDerivs*** processes.
(2) The local memory module stores the matched occupancy grid map and the LiDAR points obtained by scanning frames of the LiDAR sensor.
(3) The score/K&H matrix calculation module is the core calculation unit of the accelerator, including derivative and coordinate calculator, Grid map read controller, matrix multiplier, gradient calculator, and matrix MAC unit.

The score/K&H matrix calculation module can be used to calculate both the score under a certain pose in the CSM-based coarse match process and the Gauss–Newton iteration of a certain pose in the NLO-based fine match process, so the operation speed of the matrix calculation module affects the overall calculation speed of the accelerator and, ultimately, determines the acceleration performance of the NLO-CSM algorithm. The proposed accelerator design adopts the strategy of matrix splitting and pipeline calculation, in the hardware implementation of the K and H matrix calculation part, to achieve fast computing. Furthermore, by exploiting the operator sharing between the two-step algorithm computation (i.e., CSM and NLO algorithms), module reusing technique is also adopted to further reduce the hardware overhead of the proposed hardware accelerator.

The data processing flow of the proposed NLO-CSM hardware accelerator is described in the following discussion. The initial pose from the LiDAR sensor is sent into the preprocessing module, and the pose updater performs the storage update of the pose. The angle calculator computes the sinθ and cosθ in the function ***get_score*** and ***get_HessianDerivs*** by a CORDIC (Coordinate Rotation Digital Computer) hardware unit [18]. For the score/K&H matrix calculation module, there are two operation modes for accelerating CSM and NLO algorithms, respectively: during the CSM-based coarse match process, the grid probability value of each scan point is read from the local memory module to compute the summation of probability value of the corresponding grids and return the pose with the maximum score, according to line 12–16 in Algorithm 2, so a rational initial pose for the non-linear optimization algorithm is computed; for the NLO-based fine match process, the calculations shown in line 25–29 of Algorithm 2 can also be implemented in the score/K&H matrix calculation module. Firstly, the score/K&H matrix calculation module receives the pose information and the trigonometric calculation results from the Preprocessing module, and then, it calculates the coordinates of the LiDAR points. After calculating the addresses of the grids, according to Equations (3) and (4), the grid map data could be read out from the local memory module. Finally, the score/K&H matrix calculation module completes the calculation of K and H matrix, as described in Algorithm 2, and the results of K and H matrix would be sent to the processing system for further processing.

**Figure 10:**
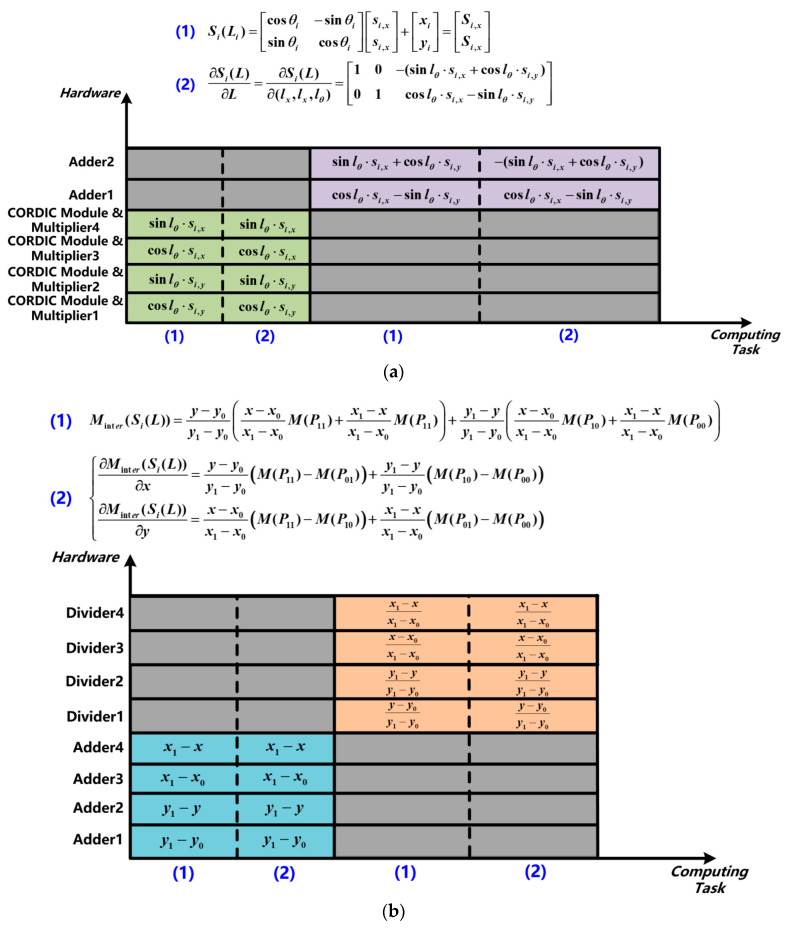
Hardware reuse scheme in (**a**) Subtask 1 and (**b**) Subtask 3 of the proposed NLO-CSM hardware accelerator design.

To realize fast computing of the NLO algorithm, this proposed accelerator design adopts the pipeline processing by segmenting the computation task of K&H matrix calculation into five subtasks and mapping the subtasks into the score/K&H matrix calculation module. By analyzing the computational load and type of tasks in the K&H matrix calculation, the segmentation of subtasks is depicted in Table 2:

(1) According to Equation (1), the calculation of $\frac{\partial S_{i}\left(L\right)}{\partial L}$ and $S_{i}\left(L\right)$ in the function ***get_score*** and ***get_HessianDerivs*** shares the same trigonometric functions and multiplication calculations, and it is segmented into subtask 1. The same hardware circuit in the score/K&H matrix calculation module can be reused to reduce the repeated calculation and hardware overhead, as shown in Figure 10a.
(2) In the local memory module, the two-dimensional grid map is stored in the one-dimensional form. The values of four LiDAR points in the grid map need to be read at a time, so the access to the local memory is segmented into subtask 2.
(3) As shown in Figure 10b, both of $\nabla M_{\mathrm{int}er}\left(S_{i}\left(L\right)\right)$ and $M_{\mathrm{int}er}\left(S_{i}\left(L\right)\right)$ use the same input data, and the calculation of relevant coordinates are consistent. Therefore, the same operation can be reused to reduce the repeated computation and hardware overhead, and it is segmented into subtask 3.
(4) The small size matrix multiplication calculation is set as subtask 4, which finishes the calculation of $j_{i}=\nabla M_{\mathrm{int}er}\left(S_{i}\left(L\right)\right)\cdot \frac{\partial S_{i}\left(L\right)}{\partial L}$.
(5) The matrix multiplication and summation of the H and K matrix is segmented into subtask 5.

Note that, for the calculation of the CSM-based coarse match process, the subtask 1 and subtask 2 calculate the address of the grid map, according to the coordinates of the LiDAR point, and find the probability value of the corresponding grids, and then, only subtask 5 completes the score calculation.

**Figure 11:**
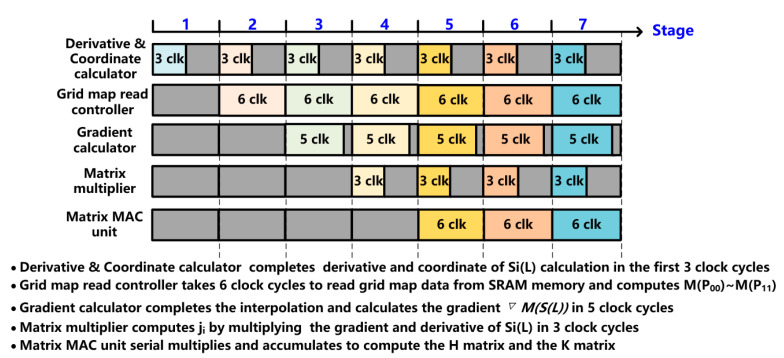
Pipeline diagram of the proposed NLO-CSM hardware accelerator.

The 5-stage pipeline diagram of the proposed NLO-CSM hardware accelerator is shown in Figure 11. In the 1st pipeline stage, the Derivative and Coordinate calculator performs the 1st subtask of $\frac{\partial S_{i}\left(L\right)}{\partial L}$ and $S_{i}\left(L\right)$ calculation by the first 3 clock cycles, and it passes the coordinate result to the grid map read controller. During the next 6 clock cycles in the 2nd pipeline stage, the grid map read controller computes the corresponding address based on the coordinates and reads grid map data from the SRAM in the local memory module. In the 3rd pipeline stage, the gradient calculator uses the grid map data to compute $\nabla M_{\mathrm{int}er}\left(S_{i}\left(L\right)\right)$ in 5 clock cycles and, then, sends the gradient result to the matrix multiplier. In the 4th pipeline stage, the matrix multiplier performs the multiplication of the gradient and derivative of *Si(L)* by 3 clock cycles. In the 5th pipeline stage, the matrix MAC unit performs the serial multiplication accumulation to compute the H and K matrix. Although the pipeline hardware utilization does not achieve a full 100%, the proposed NLO-CSM hardware accelerator employs the 5-stage pipeline scheme to effectively improve the processing throughput of computing tasks on LiDAR point stream, thus achieving fast computing speed. The pipeline latency of the proposed NLO-CSM hardware accelerator is 30 clock cycles, and the processing speed of the LiDAR point stream is only 6 clock cycles per LiDAR point.

### 3.2. Architectures of Subunits

**Figure 12:**
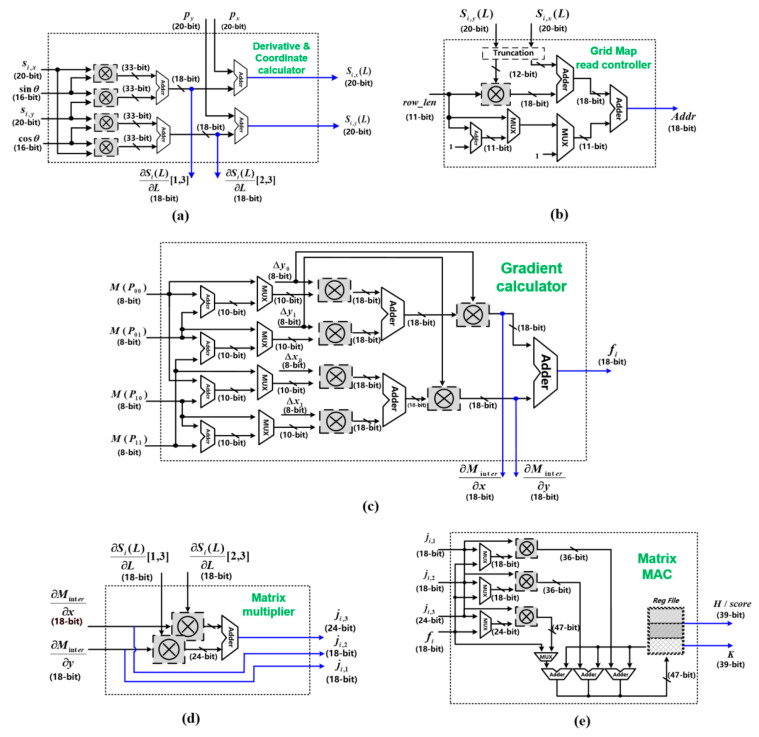
Hardware architectures of (**a**) Derivative and Coordinate calculator; (**b**) grid map read controller; (**c**) gradient calculator; (**d**) matrix multiplier; (**e**) matrix MAC unit in the proposed NLO-CSM hardware accelerator design.

The hardware architectures of the above five sub-circuit units are presented in Figure 12. As shown in Figure 12a, the Derivative and Coordinate calculator corresponds to the Derivative and Coordinate Calculation process in Figure 5, which is realized by four multipliers and four adders. The four multipliers calculate $\sin l_{\theta }\cdot s_{i,x}$, $\cos l_{\theta }\cdot s_{i,y}$, $\cos l_{\theta }\cdot s_{i,x}$, and $\sin l_{\theta }\cdot s_{i,y}$, and then, the four adders calculate the derivatives according to line 26 in Algorithm 2.

The grid map read controller calculates the address of the corresponding grid map in the local memory, according to the coordinates of the LiDAR point, as shown in Figure 12b. Specifically, due to the bilinear interpolation of the grid map, as shown in Figure 4, the coordinates of the LiDAR points and the probability values of the corresponding grids of the three surrounding points are required. The coordinates of these four points can be obtained according to the way the map is stored in the local memory, as shown in Equations (5)–(8).

(5) $$Addr\left(P_{00}\right)=S_{i}{\left(L\right)}_{y}\cdot row\_len+S_{i}{\left(L\right)}_{x}$$

(6) $$\;Addr\left(P_{10}\right)=S_{i}{\left(L\right)}_{y}\cdot row\_len+S_{i}{\left(L\right)}_{x}+1$$

(7) $$Addr\left(P_{01}\right)=S_{i}{\left(L\right)}_{y}\cdot row\_len+S_{i}{\left(L\right)}_{x}+row_{len}$$

(8) $$Addr\left(P_{11}\right)=S_{i}{\left(L\right)}_{y}\cdot row\_len+S_{i}{\left(L\right)}_{x}+row\_len+1$$

The gradient calculator completes the bilinear interpolation fitting of discrete grids to obtain the probability value and gradient of the LiDAR point, as shown in Figure 12c. The probability values *M(P*_00_*)*, *M(P*_01_*)*, *M(P*_10_*),* and *M(P*_11_*)* are multiplied with (*y − y*_0_) and (*x − x*_0_), respectively, by the MUX and multiplier, according to Equation (3). Then, the products are added and the results are multiplied with (*y − y*_0_) and (*y*_1_ *− y*). Finally, the interpolated values *f_i_* are obtained from the last adder.

The matrix multiplier performs gradient and derivative multiplication, as shown in Figure 12d. Note that, in the derivative $\frac{\partial S_{i}\left(L\right)}{\partial L}$, part of the data is constant 0 or 1, so the multiplication of the derivative and gradient can be expressed by Equation (9). There are two multipliers and adders that are utilized to calculate *j_i,_*_3_, while *j_i,_*_1 *~*_ *j_i,_*_2_ are obtained directly from the input.

(9) $$\left(\frac{\partial M_{\mathrm{int}er}\left(S_{i}\left(L\right)\right)}{\partial x},\frac{\partial M_{\mathrm{int}er}\left(S_{i}\left(L\right)\right)}{\partial y}\right)\left[\begin{array}{ccc} 1 & 0 & \frac{\partial S_{i}\left(L\right)}{\partial L}\left[1,3\right]\\ 0 & 1 & \frac{\partial S_{i}\left(L\right)}{\partial L}\left[2,3\right] \end{array}\right]=\left(j_{i,1},j_{i,2},j_{i,3}\right)$$

(10) $$H=H+j_{i}^{T}j_{i}\;\;\;\;K=K+j_{i}^{T}f_{i}$$

The architecture of the matrix MAC unit is shown in Figure 12e. The matrix MAC unit includes three multipliers, three adders, and the Reg File that stores H and K matrices. There are three multipliers that are utilized to finish the calculation of *j_i_^T^j_i_* and *j_i_^T^f_i_,* according to Equation (10), and three adders perform serial accumulation to calculate the H and K matrix. The Reg File is divided into three parts, which store the diagonal data of the H matrix, the remaining data of the H matrix, and the data of the K matrix, respectively.

## 4. Implementation Results and Discussion

This section presents the results of the proposed NLO-CSM hardware accelerator based on both Xilinx’s Zynq-7020 FPGA and 65 nm ASIC implementations. In the NLO-CSM algorithm, the number of Gauss–Newton iteration times *λ* is set to 50, the number of LiDAR point *n* is set to 90, and the down-sampling rate of the CSM-based coarse match process *SR* is set to 4 in this study. The software used for algorithm-level fixed-point modeling and simulation is Matlab; the platform used for RTL design and simulation is Xilinx’s Vivado; the tool used for ASIC simulation is Mentor’s Modelsim; the tool used for ASIC synthesis is Synopsys’s Design Compiler; the tool used for ASIC layout place and route is Cadence’s Innovus.

### 4.1. FPGA Implementation and Evaluation

The proposed NLO-CSM hardware accelerator is implemented on a Xilinx’s Zynq-7020 FPGA device. The data used to calculate the trigonometric functions, sin and cos, of the pose angle is represented in a 15-bit fixed-point number. The map coordinates, interpolation, gradient, and matrix operations of the LiDAR point are all represented in an 8-bit fixed-point number. In order to verify the proposed hardware accelerator fixed-point design, this study uses the test scenario provided by our collaborator from Shanghai University to simulate the NLO-CSM hardware accelerator, and it compares the results with the CPU software floating-point algorithm model.

As the proposed NLO-CSM hardware accelerator is designed to mainly accelerate the computation of H and K matrices by the Gauss–Newton method in the fine match process, the fixed-point results of H and K matrices are evaluated in terms of computation error. By using 200 different poses as the initial value of the fine match process to calculate the corresponding H and K matrices, this study uses the normalized root mean square error (NRMSE) to evaluate errors of the NLO-CSM hardware accelerator against the CPU software results. The NRMSEs of the H and K matrices are defined by Equations (11) and (12), respectively.

(11) $$NRMSE\left(H\right)=\frac{1}{M-N}\sqrt{\sum_{i=1}^{3}\sum_{j=1}^{3}{\left(H_{i,j}^{float}-H_{i,j}^{fixed}\right)}^{2}},M=\max(H^{float})\;\;\;N=\min(H^{fixed})$$

(12) $$NRMSE\left(K\right)=\frac{1}{M-N}\sqrt{\sum_{j=1}^{3}{\left(K_{i}^{float}-K_{i}^{fixed}\right)}^{2}},\;M=\max(K^{float})\;\;\;N=\min(K^{fixed})$$

**Figure 13:**
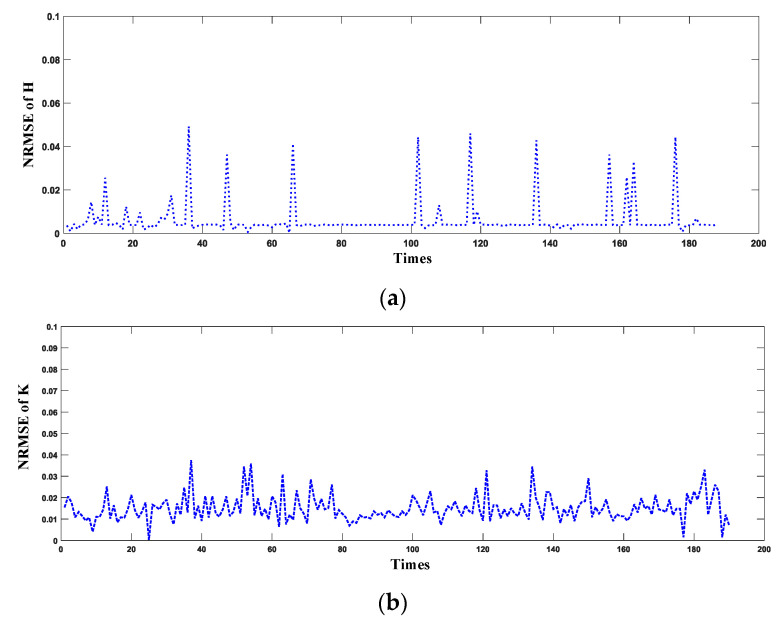
The NRMSE results of (**a**) H matrix and (**b**) K matrix computed by the proposed NLO-CSM hardware accelerator design.

The NRMSE results of H and K matrices for the fine match process under 200 different poses are shown in Figure 13. The NRMSE of the H matrix is less than 0.05, and the NRMSE of the K matrix is less than 0.04, which meets the accuracy requirements for the H and K matrices of the 2D LiDAR SLAM, using the NLO-CSM algorithm as the scan matching algorithm.

Table 3 presents the implementation results of resource utilization and power consumption of the proposed design on the Xilinx’s Zynq-7020 FPGA device. At 100 MHz clock, the proposed FPGA design performs a scan matching at 8.98 ms and 7.15 mJ. It outperforms the CPU (Arm-A9*2 @650 MHz) computation of a floating-point software, design with a scan matching at 123.76 ms and 76.98 mJ, by a 92.74% increase and 90.71% saving in computing speed and energy consumption, respectively. This result means a significant improvement in energy efficiency for the scan matching in 2D LiDAR SLAM.

**Figure 14:**
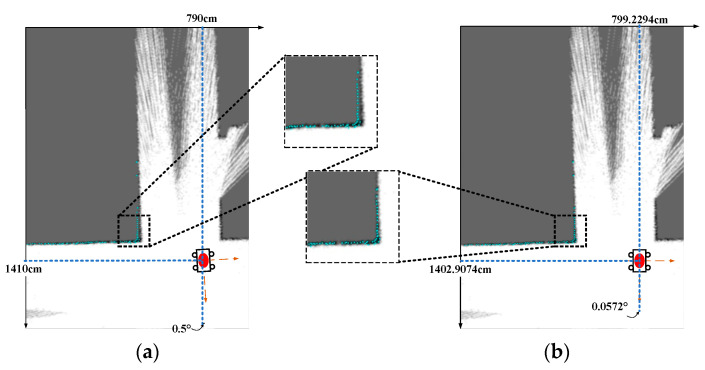
Mapping between scan points and grid map under the (**a**) initial pose; (**b**) the pose from the proposed NLO-CSM hardware accelerator.

Figure 14 shows the scan matching result of an indoor corner in a grid map (i.e., a 10 m × 16 m rectangle) whose resolution is 5 cm. The initial pose value is (790 cm, 1410 cm, 0.5°). Figure 14a shows the mapping between scan points and the grid map under the initial pose, while Figure 14b shows the matching result of (799.2294 cm, 1402.9074 cm, 0.0572°) calculated by the proposed hardware accelerator. The baseline result, calculated by a floating-point software design, is (799.2798 cm, 1402.8616 cm, 0.0594°). The error of matching results of the proposed accelerator is around 0.05 cm and 0.002°, which is negligible at 5 cm resolution, meaning there is a high matching accuracy.

**Figure 15:**
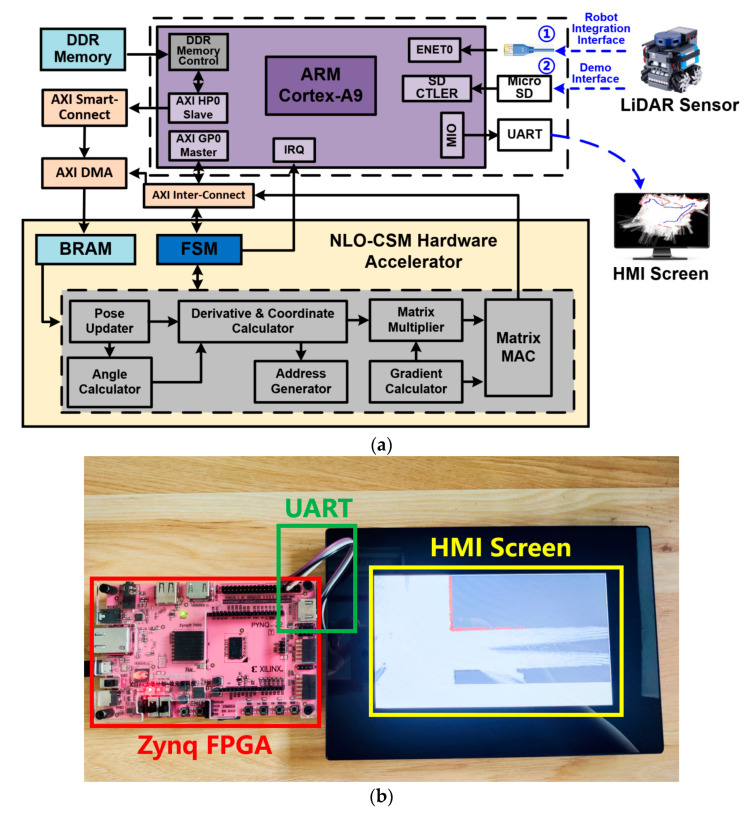
(**a**) SoC architecture and (**b**) a photo of the proposed NLO-CSM hardware accelerator’s demo for scan matching of an indoor corner based on a Zynq FPGA kit.

As shown in Figure 15a, this work uses a Xilinx’s Zynq-7020 FPGA device to realize the SoC system of the NLO-CSM hardware accelerator, and it realizes the overall accelerated calculation of NLO-CSM algorithm through software and hardware co-design. When the SoC system initiates, the Processing System (PS) of the FPGA writes control commands to the AXI DMA module through the AXI-Lite bus pathway, and it sends commands to the NLO-CSM hardware accelerator to write grid map and LiDAR frame data. Then, the accelerator enters the state of receiving data. After that, the AXI DMA module completes the transfer of grid map and LiDAR frame data to BRAM through the AXI-Stream data path, and the PS sends the calculation instruction to the hardware accelerator through the AXI-Lite bus path, and subsequently, the accelerator starts the calculation task. When the accelerator completes the acceleration task, it sends the interrupt signal through the IRQ port of the PS side, and the PS will read the final result through the AXI-Lite bus path. Finally, the PS sends the matching results to the HMI screen through UART port for a result demonstration. The demo photo of the proposed NLO-CSM hardware accelerator SoC system’s result for the scan matching of a corner is shown in Figure 15b.

### 4.2. ASIC Implementation and Discussion

**Figure 16:**
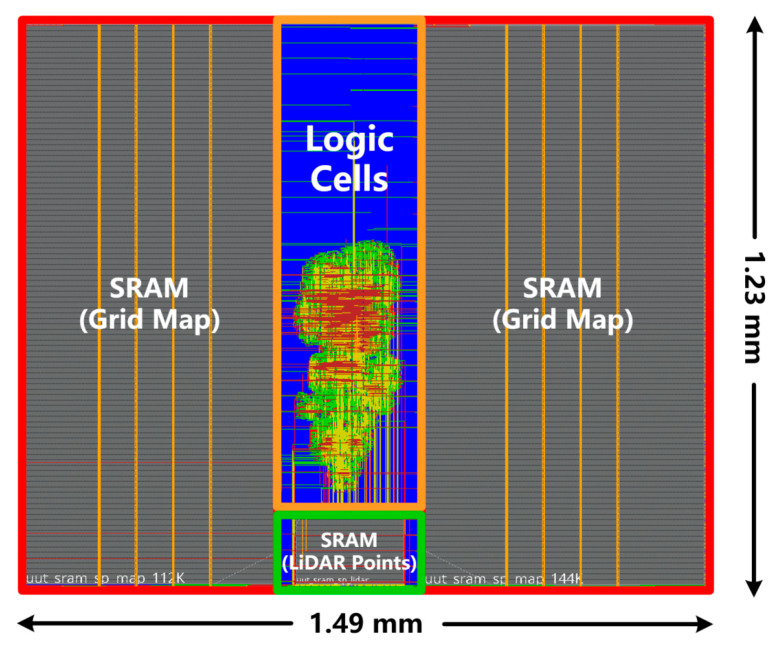
ASIC layout of the proposed NLO-CSM hardware accelerator core in 65 nm process.

In this study, the proposed NLO-CSM hardware accelerator is also implemented in a 65 nm CMOS process node, and the ASIC layout is shown in Figure 16. Note that, because of relatively low data throughput from a single-line LiDAR sensor, this work adopts high-density single-port SRAM in the ASIC implementation to realize the storage of local memory for the grid map and LiDAR points to reduce hardware overhead. The 256-KB SRAM is used for storing the grid map, while 3-KB SRAM is used for buffering LiDAR points. The ASIC implementation results of the proposed NLO-CSM hardware accelerator core are summarized in Table 4.

**Figure 17:**
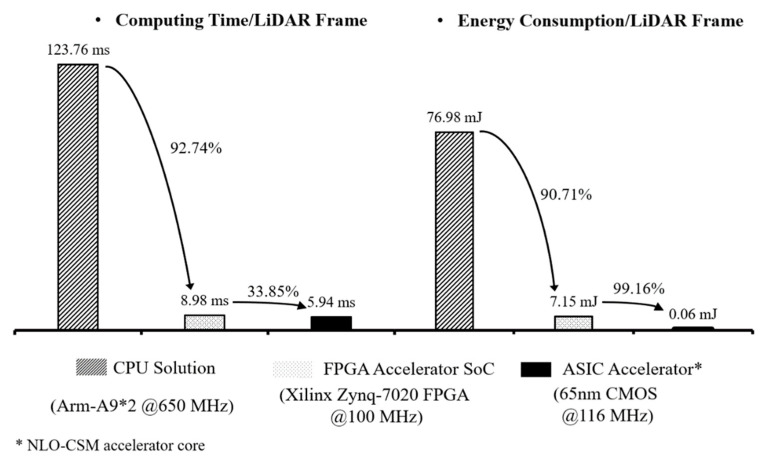
Comparison of computing time and energy consumption, per LiDAR frame, among the CPU software solution, FPGA hardware accelerator core, and ASIC accelerator core of the NLO-CSM algorithm.

Figure 17 presents the comparison of different implementation solutions of the NLO-CSM algorithm. Compared with the FPGA results, the ASIC implementation of the proposed NLO-CSM hardware accelerator design reduces the computing time and the energy consumption, per LiDAR frame, to 5.94 ms and 0.06 mJ at the maximum clock rate of 116 MHz, respectively, which shows that faster computing and higher energy efficiency can be achieved by the ASIC-based hardware accelerator core design of scan matching in 2D LiDAR SLAM for mobile and micro robot applications.

### 4.3. Comparison with the State-of-the-Art and Discussions

Table 5 presents the performance evaluation results of the proposed FPGA-based NLO-CSM accelerator SoC (System-on-Chip) against the state-of-the-art designs in the literature. The design in [16] is based on the conventional CSM, while the design in [17] is based on a complicated RIA-CSM that requires significantly more hardware resources in terms of computation logic and storage memory. As compared to the design in [17] that has an impact-aware feature to implement real-time robot impact detection, our proposed NLO-CSM accelerator design is less robust to robot impact. As the NLO-CSM adopted in this work is actually based on the conventional CSM algorithm, the conventional CSM hardware accelerator design in [16] is chosen for a fair comparison in this study.

For the hardware resources, the proposed NLO-CSM accelerator design has saved 80.3% LUTs, 84.13% FFs, and 20.83% DSPs against the hardware accelerator of the conventional CSM in [16], thanks to the adopted low-computation NLO-CSM algorithm, as well as the proposed module reuse technique. For the processing speed, benefiting from the low-computation algorithm and the adopted pipeline processing scheme, a frame rate of 111.29 fps has been achieved by the proposed design at the same 100 MHz clock, i.e., 8.17× higher than the design in [17]. For energy efficiency, the proposed design has achieved 7.15 mJ/frame, which is 96.22% lower than the design in [16]. The improvement of energy efficiency is benefited from two aspects. In one hand, the adopted NLO-CSM algorithm has a much lower amount of computation that results in lower computation power and shorter computation time. In the other hand, the pipeline processing has further improved the processing speed of the proposed hardware accelerator.

In summary, the comparison results show that the proposed NLO-CSM accelerator design has achieved significantly lower hardware resource consumption and higher energy efficiency, while ensuring a much higher frame rate, as compared to the state-of-the-art designs. This study indicates that the proposed NLO-CSM accelerator design is more suitable for resource-limited and energy-constrained mobile and micro robot applications.

## 5. Conclusions

This paper proposes an efficient hardware accelerator design of the NLO-CSM algorithm for scan matching in 2D LiDAR SLAM. An optimized NLO-CSM algorithm is adopted in this work to reduce the computation resources and the amount of computation, as well as avoid the high computational complexity of brute force searching on the grid map in the conventional CSM algorithm, while maintaining a good scan match performance. By exploiting the algorithm’s similarity and operator sharing between the two-step algorithm computations, the module reusing technique is adopted to further reduce the hardware overhead, and the pipeline processing scheme is also adopted for fast computing, therefore achieving high energy efficiency. The FPGA implementation results, based on Xilinx Zynq-7020 FPGA, show that the proposed hardware accelerator has achieved a 92.74% increase and 90.71% saving in computing speed and energy consumption per frame, as compared to the conventional ARM-A9 dual-core CPU implementation. Compared with a state-of-the-art design, the proposed hardware accelerator has achieved 80.3% LUTs, 84.13% FFs, and 20.83% DSPs saving, as well as 8.17× increase in frame rate and a 96.22% improvement in energy efficiency. The 65 nm ASIC implementation result of 5.94 ms and 0.06 mJ per frame is a further improvement in both the scan speed and energy efficiency, which shows that the proposed NLO-CSM hardware accelerator design is suitable for the resource-limited and energy-constrained intelligent mobile and micro robot applications.

## Figures and Tables

**Table 1:** Comparison of the localization error between the adopted NLO-CSM and the RIA-CSM.

Dataset	Error Type	Localization Error	
		This Work	IEEE Sensors Journal’ 2022 [15]
**Deutsches Museum**	Abs translational error (m)	0.02332 ± 0.01634	0.03034 ± 0.02193
	Sqr translational error ($m^{2}$)	0.00081 ± 0.00155	0.00140 ± 0.00241
	Abs rotational error (deg)	0.14480 ± 0.13388	0.15550 ± 0.15115
	Sqr rotational error (deg^2^)	0.03889 ± 0.08288	0.04687 ± 0.09481
**Revo LDS**	Abs translational error (m)	0.02092 ± 0.02595	0.03185 ± 0.01916
	Sqr translational error ($m^{2}$)	0.00111 ± 0.00299	0.00138 ± 0.00156
	Abs rotational error (deg)	0.23718 ± 0.23081	0.22776 ± 0.16350
	Sqr rotational error (deg^2^)	0.10941 ± 0.19017	0.07839 ± 0.09769

**Table 2:** Segmentation of Computation Tasks in the Fine Match Process.

	Computing Task	Hardware Module
Subtask 1	$\frac{\partial S_{i}(L)}{\partial L}$, $S_{i}(L)$	Derivative & Coordinate calculator
Subtask 2	$\begin{array}{cccc} M(P_{11}) & M(P_{01}) & M(P_{10}) & M(P_{00}) \end{array}$	Grid map read controller
Subtask 3	$\nabla M_{\mathrm{int}er}(S_{i}(L))$, $f_{i}=1-M_{\mathrm{int}er}(S_{i}(L))$	Gradient calculator
Subtask 4	$j_{i}=\nabla M_{\mathrm{int}er}(S_{i}(L))\cdot \frac{\partial S_{i}(L)}{\partial L}$	Matrix multiplier
Subtask 5	$H=H+j_{i}^{T}j_{i}$ $K=K+j_{i}^{T}f_{i}$	Matrix MAC unit

**Table 3:** Resource Utilization and Power Consumption of the Proposed Hardware Accelerator SoC Design on a Xilinx Zynq FPGA.

LUT	4142
FF	3193
DSP	19
BRAM	297 KB
Power	0.79 W@100 MHz

**Table 4:** ASIC Implementation Results of the Proposed NLO-CSM Hardware Accelerator Core.

Process	65 nm
Area	1.49 × 1.23 mm^2^
Supply Voltage	1.08 V
Gates	1.37 M
Memory	259 KB
Power	11.2 mW@116 MHz

**Table 5:** The proposed NLO-CSM accelerator, based SoC’s characteristics and performance evaluation results, compared to the state-of-the-arts.

Publication	IEEE Access’2022 [16]	IEEE Sensors Journal’2022 [17]	This Work
FPGA Platform	Zynq-7020 (SoC) (28 nm FPGA)	AX7Z100 (SoC) (28 nm FPGA)	Zynq-7020 (SoC) (28 nm FPGA)
Algorithm	Conventional CSM	RIA-CSM (Real-Time Impact-Aware CSM)	NLO-CSM
Abs translational error (m)	0.0376 ± 0.0307 (Based on MIT-CSAIL dataset)	0.03185 ± 0.01916 (Based on Revo LDS dataset)	0.02092 ± 0.02595 (Based on Revo LDS dataset)
Grid Resolution (cm)	5	5	5
Frequency (MHz)	100	133	100
LUTs	21026	13870	4142
FFs	20121	22747	3193
BRAM (KB)	444	1962.5	297
DSPs	24	32	19
Frame Rate (fps)	12.13 @100 MHz	89.58 @133 MHz (67.19 @100 MHz)	111.29 @100 MHz
Power Consumption	2.3 W @100 MHz	2.113 W @133 MHz (1.58 W @100 MHz)	0.79 W @100 MHz
Energy Efficiency	189.52 mJ/frame	23.58 mJ/frame	7.15 mJ/frame
